# The Prevalence of Specific Learning Difficulties in Higher Education: A Study of UK Universities Across 12 Academic Years

**DOI:** 10.1177/00222194241281479

**Published:** 2024-10-20

**Authors:** Nicola Brunswick, Nathan J. Wilson, Ian Kruger, Rebecca Chamberlain, I. Christopher McManus

**Affiliations:** 1 Middlesex University, London, UK; 2 Goldsmiths, University of London, UK; 3 University College London, UK

**Keywords:** specific learning difficulties, ADHD, prevalence, higher education (HE), academic subjects, ethnicity

## Abstract

Specific learning and attention difficulties are often first identified in childhood, but they can cause lifelong academic and occupational challenges. We explored the prevalence of these difficulties and the representation of sex and ethnicity amongst all first-year students in United Kingdom (UK) higher education (HE) across 12 years—almost 5.7 million students—and compared course preferences and university destinations of those with and without difficulties. Students declaring learning/attention difficulties were more likely to be White or of Mixed ethnicity and least likely to be Asian. They were more likely to attend specialist HE institutions or newer universities, and more likely to study courses in creative arts and design, agriculture and architecture than law, languages, computer science, and mathematical sciences. The number of students declaring difficulties has increased year on year, in actual terms and as a proportion of the student body, suggesting that efforts to increase diversity and inclusion have been successful. However, differences remain between students with and without learning/attention difficulties in terms of ethnicity, subjects studied, and HE institutions attended, so more needs to be done to identify and address reasons for this. While this paper reports data from UK students, it addresses an international question and invites similar explorations of other national datasets.

Specific learning difficulties (SpLD) are neurodevelopmental disorders that affect a person’s ability to learn, particularly affecting their reading, writing, and mathematical ability. Difficulties exist on a continuum from mild to severe, and they frequently co-occur; many individuals with SpLD also have difficulty with inattention and/or hyperactivity (British Dyslexia Association, 2018). Effects are often first identified in childhood, but they can continue to impact academic and occupational decisions and achievements across the lifetime (Chatzitheochari & Platt, 2019; Parsons & Platt, 2022; see also Shrewsbury, 2015). For example, students with SpLD have been found to be significantly less likely than non-SpLD students to enter higher education (HE) by age 19 (Chatzitheochari & Platt, 2019, GOV.UK, 2021). Students with SpLD who do enter HE are less likely to complete their studies or, if completed, to achieve a first-class (70%+) or upper second-class (60–69%) grade (Bolton & Lewis, 2023; Pumphrey, 2008; Richardson, 2015).

In the United Kingdom, between 1994/95 (the earliest year for which data are available in the Higher Education Statistics Agency [HESA] archive) and 2004/05, the number of first-year undergraduate and postgraduate students who declared an SpLD on their university application increased from 2,359 (0.4% of students) to 21,000 (2.5% of students) (HESA, 2006). A decade later (2014/15), the number was 106,595 (5.7% of students in HE institutions; HESA, 2021). Until the academic year 2006/07, HESA listed dyslexia as a distinct category of difficulty, but from 2007/08 onwards it was subsumed into a broader category—‘A specific learning difficulty such as dyslexia, dyspraxia or ADHD’^1^—which would account for some, but certainly not all, of this increase.

Data also suggest that students with SpLD may elect to study some university courses more frequently than not. Students with dyslexia, for example, are disproportionately present in disciplines related to art and design (Bacon & Bennett, 2013; Holgate, 2015; Wolff & Lundberg, 2002). Small but significant increases in numbers of students with SpLD have been found in nursing and midwifery (Sanderson-Mann & McCandless, 2005; Taylor & Walter, 2003), dentistry (Cruikshank et al., 2002), and other subjects allied to medicine (Yeowell et al., 2015).

In the largest previous study, Richardson and Wydell (2003) analyzed national student data from the 1995–96 academic year. They found that students with SpLD (0.46% of all students) were most likely to study agriculture, creative arts, engineering, architecture, physical sciences, humanities, social studies, biological sciences, and computer science, but least likely to study veterinary science, languages, law, education, medicine, and subjects allied to medicine. Richardson (2015) found that 4.97% of one cohort of first-year UK students reported SpLD and were more likely to study health, social care, and social sciences, but least likely to study business and law.

Outside the United Kingdom, differences in degrees pursued by students with SpLD are also found. A study of Greek students (*N* = 251,433) at 32 public higher education institutions (HEIs) found that those with dyslexia were most likely to study technological science, business administration, and agricultural science, and least likely to study health subjects, social sciences, or the arts (Stampoltzis & Polychronopoulou, 2008). A study of Italian students across 19 public universities (*N* = 585) found that those who had used the university learning support service within the previous year (0.13% of the student body) were most likely to study statistics, agriculture, veterinary science, education, and architecture, and least likely to study economics, engineering, medicine, law, languages, and sport science (Longobardi et al., 2019).

However, although students appear more likely to study agriculture and architecture, and less likely to study languages, law, and medicine/subjects allied to medicine (Richardson & Wydell, 2003; Stampoltzis & Polychronopoulou, 2008), Longobardi et al. (2019) found that students with SpLD were most likely to study education and veterinary science. Conversely, Richardson and Wydell (2003) found these subjects least likely to be studied. While Richardson and Wydell (2003) found engineering and social sciences to be more likely to be studied by SpLD students, others found them least likely (Longobardi et al., 2019; Stampoltzis & Polychronopoulou, 2008). Similarly, Richardson (2015) found high numbers of students with SpLD studying health, while Stampoltzis and Polychronopoulou (2008) found low numbers, inconsistencies, which might be explained by differences in sample sizes, criteria, and recruitment differences in SpLD.

There also appear to be differences in the types of institution chosen by applicants with SpLD. Using UK data, Singleton (1999) found lower numbers of students with dyslexia in “pre-92” UK universities (established prior to the Further and Higher Education Act 1992, these have tended to be more selective and require higher entrance qualifications) than in “post-92” universities (former polytechnics and colleges that were given university status in/since 1992, these have tended to be less selective, more likely to accept students from nontraditional academic backgrounds, and to offer more applied, practical and vocational courses). The largest numbers were in specialist HEIs (institutions offering courses solely in one specialist area such as art, drama, or music), between 1.54% and 6% of students (Riddell et al., 2005; Singleton, 1999). Another distinction might be drawn between “Russell Group” and non-Russell Group universities. The Russell Group refers to an organization of 24 universities that are highly selective (requiring high entry qualifications), research-focused, and accept a smaller percentage of students through clearing^2^ than do other pre-92 or post-92 universities (Hemsley-Brown, 2015; Raffe & Croxford, 2015). Richardson (2009) reported that university students with dyslexia were less likely to attend Russell Group universities than other HEIs.

Previous studies of students with SpLD at university have collected data individually from HEIs responding to questionnaires (Longobardi et al., 2019; Stampoltzis & Polychronopoulou, 2008), from a single HEI (Richardson, 2015) or a single academic year (Richardson, 2009; Richardson & Wydell, 2003). The result of this variety of methodological approaches is that some consistencies might be overlooked (or over-represented), or that more inconsistencies than truly exist may be reported.

The current study, therefore, sought to provide a comprehensive analysis of the role of SpLD/ADHD in course and institution selection via analysis of data from all students on all courses leading to recognized qualifications or academic credit at all publicly funded UK institutions over 12 years. Data were drawn from the Higher Education Information Database for Institutions (HEIDI) which includes quantitative data about HE (staff, students, finances, estates) and developed by the United Kingdom’s Higher Education Statistics Agency (www.hesa.ac.uk). In the United Kingdom, there is a statutory obligation on the 167 government-funded higher-level academic institutions to return data to HESA regarding all enrolled students. Data submitted include age, sex, ethnicity, domicile, self-reported disabilities, highest qualification on entry, course of study, university attended, and qualification awarded. Data are collected by census and subject to audit by HESA; they are compared against data returned to funding bodies to mitigate against inaccuracies, so we can be confident that this is as accurate a record as possible. However, disability data are based on student self-report rather than evidence of formal assessment which may be requested later by universities to enable students to access specialist support. Therefore, only students who declare SpLD/ADHD on their university application or when they register with a university will be recorded.

In return for universities returning data to HESA, these data are compiled and made available for academic research subject to the requirements of the General Data Protection Regulation and other relevant UK data protection legislation under the Creative Commons Attribution 4.0 International (CC BY 4.0) license (https://creativecommons.org/licenses/by/4.0/).

Using this database, we sought to answer the following questions: What percentage of UK students declare an SpLD/ADHD on their university application? Has this changed over the 12 years covered by this study? Is there a relationship between SpLD/ADHD and sex or ethnicity? Are any subjects more frequently studied by students with SpLD/ADHD? Do students with SpLD/ADHD choose different types of universities than students without SpLD/ADHD? Are SpLD/ADHD students more likely to choose pre-92 or post-92 universities, Russell Group or non-Russell Group universities, or more specialist institutions? Answers to these questions could provide valuable insight into how SpLD/ADHD influences the HE choices and opportunities of students. Such insight is important for making informed decisions that contribute to fostering equality, embracing diversity, and ensuring the inclusive participation of students with SpLD/ADHD in UK higher education.

## Method

Data were downloaded from the “HESA Student Qualifiers Full Person Equivalent (FPE) v1” database in March 2022 by the second author (NJW), an authorized Gold User of the Heidi Plus platform. In accordance with the user agreement, data were downloaded and analyzed in the United Kingdom solely for research purposes, the Heidi Plus software and functionality were not altered, and the Heidi Plus Rounding Methodology was applied to protect individuals’ identity. Frequency counts were rounded to the nearest multiple of five, and percentages based on fewer than 22.5 students (after any calculations) were suppressed. Therefore, totals may not match across tables, and percentages may not sum to exactly 100.

In view of the change in the way that HESA classified SpLD—from “dyslexia” up until the academic year 2006/07 to “A specific learning difficulty such as dyslexia, dyspraxia, or AD(H)D” from 2007/08 onwards—2007/08 was taken as our starting point.

We obtained data on student characteristics including sex, ethnicity, disability status, subject of study, and HEI. Under disability status, students are listed as having “no known disability”, one of eight listed disabilities (see Table 2), or “two or more impairments and/or disabling medical conditions.” Of students who declare a disability, those with “a specific learning difficulty such as dyslexia, dyspraxia, or AD(H)D” form the largest group. Within this group, around 90% are likely to have dyslexia as it is the most common SpLD (National Association of Special Education Teachers, n.d.), although there is high co-occurrence between learning difficulties (Government Office for Science, 2020). Some students with multiple difficulties will appear in the “two or more impairments and/or disabling medical conditions” category. The way that HESA data have been categorized since 2007/08 does not allow students with SpLD/ADHD to be subdivided to allow exploration of the academic choices of those with each need or combination of needs.

Data were exported into Microsoft Excel (version 16.60) for all UK-domiciled first-year students—those whose permanent home address was in the United Kingdom at the time they submitted their university applications—who were enrolled on first degree courses at UK HEIs between 2007 and 2019. Ethical approval was granted by Middlesex University’s Psychology Ethics Committee.

## Results

### Demographics

Between 2007/08–2018/19, there were 5,653,070 UK-domiciled first-year undergraduate students enrolled at UK HEIs. This is the entire population of first-year students, so statistical analysis of the data is neither necessary nor appropriate; instead, data are described, highlighting patterns within and across subgroups in the population, and confidence intervals are provided.

Table 1 shows students’ sex, ethnicity, and HEI type. Most students were White (77.70%), followed by Asian (10.23%), Black (7.17%) or Mixed ethnicity (3.63%).^3^ The sample was 56.35% female, 52.72% attended post-92 universities, and 44.26% attended pre-92 universities. In the latter group, 43.97% attended Russell Group universities. A minority (3.02%) attended specialist institutions.

**Table 1.** Student Sex, Ethnicity, and Higher Education Institution Type Attended From 2007 to 2019 (Source: HESA)

Demographic characteristics	Number ^a^	Percentage
*Sex*:		
Female	3,153,460	56.35
Male	2,442,115	43.64
Other	420	0.01
*Ethnicity*:		
Asian	562,530	10.23
Black	394,060	7.17
Mixed	199,475	3.63
Other	70,000	1.27
White	4,273,190	77.70
*Institution type*:		
Pre-92 university (all: *n* = 58)	2,502,010	44.26
Pre-92 non-Russell Group university (*n* = 34)	1,401,770	56.03
Russell Group university (*n* = 24)	1,100,240	43.97
Post-92 university (*n* = 71)	2,980,160	52.72
Specialist institution (*n* = 34)	170,900	3.02

a Excluding those for whom sex/ethnicity was not known.

*Note*. Pre-92 universities = established prior to the Further and Higher Education Act 1992, Post-92 = established after; Russell Group = 24 highly selective, research-focused universities that accept a smaller percentage of students through clearing; HESA = Higher Education Statistics Agency.

Table 2 shows the prevalence of each disability. Of the almost 5.7 million first-year undergraduates, 5.10% reported SpLD/ADHD, while 88.71% reported no known disability. The SpLD/ADHD group comprised almost half (45.22%) of those with a declared disability, almost three times the number reporting a mental health condition (16.07%) and 4.5 times the number declaring long-standing illness (10.16%).

**Table 2.** Prevalence of Disability Recorded for Students in UK Higher Education Institutions Between 2007 and 2019 (Source: HESA).

Disability (category names used by HESA):	Number	% of total	% with a disability
No known disability/unknown	5,014,990	88.71	-
A specific learning difficulty such as dyslexia, dyspraxia, or AD(H)D	288,540	5.10	45.22
A mental health condition, such as depression, schizophrenia, or anxiety disorder	102,525	1.81	16.07
A long-standing illness or health condition such as cancer, HIV, diabetes, chronic heart disease, or epilepsy	64,820	1.15	10.16
A disability, impairment or medical condition that is not listed	61,270	1.08	9.60
Two or more impairments and/or disabling medical conditions ^a^	57.075	1.01	8.94
A social communication impairment such as Asperger’s syndrome/other autistic spectrum disorder	23,860	0.42	3.74
A physical impairment or mobility issues, such as difficulty using arms or using a wheelchair or crutches	18,405	0.33	2.88
Deaf or a serious hearing impairment	14,055	0.25	2.20
Blind or a serious visual impairment uncorrected by glasses	7,500	0.13	1.18

a Students from this category were excluded from statistical analysis to avoid potential double-counting.

*Note*. HESA = Higher Education Statistics Agency.

**Figure 1.**
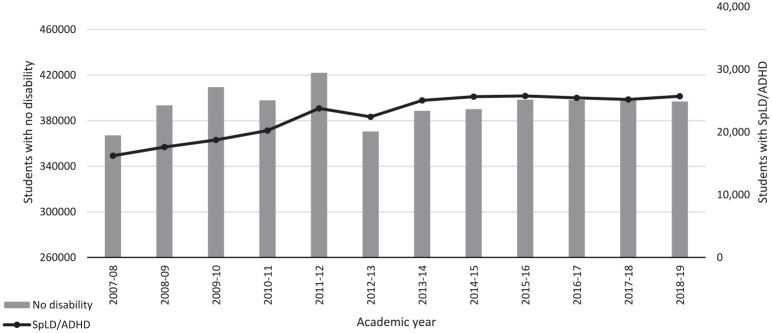
Number of First-Year Students Per Year Reporting No Disability or SpLD/ADHD (Source: HESA). *Note.* The total number of undergraduate enrolments at UK HEIs decreased by 6.4% between 2011–12 and 2012–13; this coincided with an increase in annual tuition fees from £3,375 to £9,000. SpLD/ADHD = specific learning difficulties/attention-deficit/hyperactivity disorder; HESA = Higher Education Statistics Agency.

**Figure 2.**
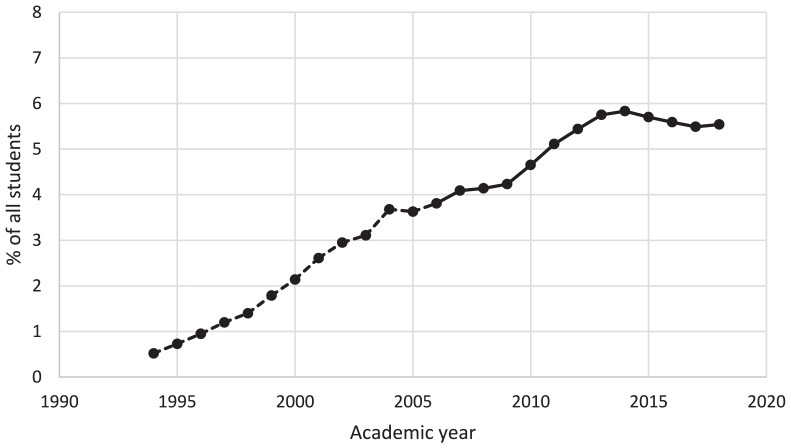
Number of First-Year Undergraduate Students Per Year Reporting Dyslexia (1994/95 to 2006/07—Dashed Line) or SpLD/ADHD (2007/08 to 2018/19—Solid Line) (Source: HESA). *Note*. SpLD/ADHD = specific learning difficulties/attention-deficit/hyperactivity disorder; HESA = Higher Education Statistics Agency.

**Figure 3.**
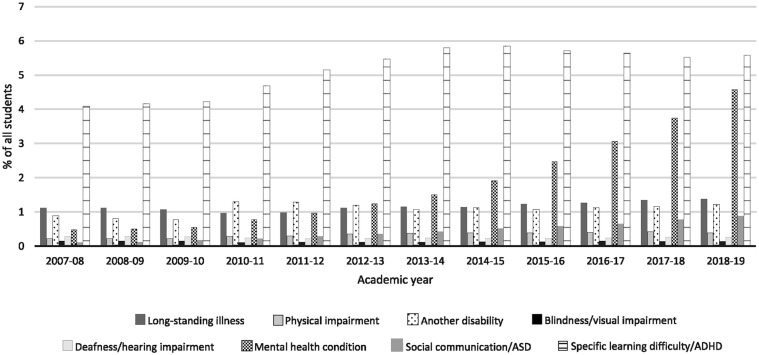
UK First-Year Higher Education Institution Students Reporting Disabilities Per Year, as a Percentage of the Total Student Body (2007–2019) (Source: HESA). *Note*. HESA = Higher Education Statistics Agency.

The number of students with SpLD/ADHD has remained fairly stable across the years covered by this study, at around 4–6% of the student body (see Table 3), although the number increased from 17,510 (4. 08%) in 2007/08 to 26,705 (5.57%) in 2018/19 (see Figure 1). The number reporting no disability increased from 397,345 in 2007/08 to 410,005 in 2018/19 although proportionally this figure reduced from 92.66% to 85.57%. To contextualize these figures, we downloaded additional data from the HESA archive (https://www.hesa.ac.uk/data-and-analysis/publications) showing numbers of first-year undergraduates reporting dyslexia from 1994/95 (the first year for which data are available) to 2006/07. These data, plus our current data, are plotted in Figure 2. As this shows, the proportion of students reporting dyslexia rose steadily from 1994/95 (0.52%) to 2004/05 (3.68%), then remained around 4% until 2010/11 (spanning the years 2006/07 to 2007/08 when the classification of dyslexia changed); the proportion of students reporting any type of SpLD/ADHD then increased until 2014/15 (5.85%) before decreasing slightly. Alongside this increase in SpLD/ADHD has been a large increase in students reporting mental health conditions, from 0.48% of all students in 2007/08% to 4.57% in 2018/19 (see Figure 3).

**Table 3.** Number of Enrolled Students With No Known/Reported Disability and Those Reporting SpLD/ADHD From 2007 to 2019 (Source: HESA).

	SpLD/ADHD		No known disability	
Academic year	Number ^a^	% within year	Number ^a^	% within year
2007/8	17,510	4.08	397,345	92.66
2008/9	19,215	4.16	427,285	92.62
2009/10	20,460	4.22	448,240	92.52
2010/11	22,160	4.68	432,570	91.40
2011/12	25,815	5.15	454,550	90.68
2012/13	23,825	5.47	391,440	89.88
2013/14	26,480	5.79	408,345	89.33
2014/15	26,920	5.85	408,010	88.70
2015/16	26,785	5.71	413,875	88.20
2016/17	26,585	5.63	412,965	87.47
2017/18	26,080	5.51	410,360	86.65
2018/19	26,705	5.57	410,005	85.57

a All counts are rounded to the nearest multiple of 5 in accordance with the Heidi Plus Rounding Methodology and other relevant data protection legislation in the United Kingdom.

*Note*. SpLD/ADHD = specific learning difficulties/attention-deficit/hyperactivity disorder; HESA = Higher Education Statistics Agency; HEIDI = Higher Education Information Database for Institutions.

More male students (5.44%, 95% CI = 5.41, 5.47) than female students (4.94%, CI = 4.91, 4.96) registered as having SpLD/ADHD, indicating a prevalence rate 1.10 times higher in male than female students (see Table 4). As there were proportionately more females than males, the number of female students with SpLD/ADHD made up a larger percentage of the student body (2.78%, CI = 2.77, 2.79 vs 2.37%, CI = 2.36, 2.39).

**Table 4.** Sex and Ethnicity of Students With Reported SpLD/ADHD and Those With No Known/Reported Disability (Source: HESA).

Demographic characteristics	SpLD/ADHD		Prevalence of SpLD/ADHD	No known disability	
	Number	(%)^a^ (95% CIs)	(%)^b^ (95% CIs)	Number	%^a^ (95% CIs)
*Sex*:					
Female	155,640	4.94 (4.91–4.96)	2.78 (2.77–2.79)	2,818,425	89.38 (89.34–89.41)
Male	132,880	5.44 (5.41–5.47)	2.37 (2.36–2.39)	2,196,225	89.93 (89.89–89.97)
*Ethnicity*:					
Asian	11,850	2.11 (2.07–2.14)	0.22 (0.21–0.22)	535,880	95.26 (95.21–95.32)
Black	16,655	4.23 (4.16–4.29)	0.30 (0.30–0.31)	363,695	92.29 (92.21–92.38)
Mixed	11,345	5.69 (5.59–5.79)	0.21 (0.20–0.21)	179,400	89.94 (89.80–90.07)
Other	2,320	3.31 (3.18–3.45)	0.04 (0.04–0.04)	66,370	94.81 (94.65–94.98)
White	240,975	5.64 (5.62–5.66)	4.38 (4.36–4.40)	3,791,870	88.74 (88.71–88.77)

*Note*. SpLD/ADHD = specific learning difficulties/attention-deficit/hyperactivity disorder; HESA = Higher Education Statistics Agency.

a Although percentages were calculated against total number of students within each group, only results for those with no known disability or SpLD/ADHD are reported. ^b^Prevalence of SpLD/ADHD was calculated for each group against total number of enrolled students.

Among those who declared their ethnicity, the distribution with SpLD/ADHD versus those without differed greatly. Higher numbers of Mixed ethnicity (5.69%, CI = 5.59, 5.79) and White students (5.64%, CI = 5.62, 5.66) reported SpLD/ADHD than did those in any other ethnic group. Students reporting SpLD/ADHD were least likely to be Asian (2.11%, CI = 2.07, 2.14). Controlling for differences in ethnic group size, White students with SpLD/ADHD formed a much larger percentage (4.38%, CI = 4.36, 4.40) than SpLD/ADHD students of any other ethnicity; Asian students with SpLD/ADHD constituted a smaller percentage (0.22%, CI = 0.21, 0.22) than expected based on UK population statistics.

### Subject of Study

As Table 5 shows, subject areas with the largest percentage of SpLD/ADHD students were agriculture and related subjects (10.08%, CI = 9.73, 10.44), creative art and design (9.69%, CI = 9.59, 9.79), architecture, building, and planning (6.76%, CI = 6.56, 6.96), subjects allied to medicine (6.35%, CI = 6.27, 6.42), and social studies (5.86%, CI = 5.78, 5.94). Smaller proportions of SpLD/ADHD students were studying business and administrative studies (4.40%, CI = 4.33, 4.46), languages (3.25%, CI = 3.16, 3.34), mathematical sciences (3.23%, CI = 3.09, 3.37), and law (3.14%, CI = 3.04, 3.23). Accounting for number of students studying each subject, those with the highest prevalence of SpLD/ADHD were: creative arts and design (0.94%, CI = 0.93, 0.96), subjects allied to medicine (0.77%, CI = 0.77, 0.78), biological sciences (0.62%, CI = 0.61, 0.63), and social studies (0.62%, CI = 0.61, 0.63). Subjects with the lowest prevalence were: medicine and dentistry (0.09%, CI = 0.09, 1.00), agriculture and related subjects (0.09%, CI = 0.08, 0.09), mathematical sciences (0.07%, CI = 0.06, 0.07), and veterinary science (0.01%, CI = 0.01, 0.01).

**Table 5.** Disciplines Selected by Individuals Reporting SpLD/ADHD, Using the Joint Academic Coding System (JACS) Subject Areas (Source: HESA).

JACS subject area:	SpLD/ADHD - %^a^ (95% CIs)	Prevalence of SpLD/ADHD^b^ (95% CIs)	No known disability - %^a^ (95% CIs)
Agriculture & related subjects	10.08 (9.73–10.44)	0.09 (0.08–0.09)	82.87 (82.43–83.32)
Creative arts & design	9.69 (9.59–9.79)	0.94 (0.93–0.96)	81.05 (80.91–81.19)
Architecture, building & planning	6.76 (6.56–6.96)	0.13 (0.12–0.13)	89.35 (89.10–89.60)
Subjects allied to medicine	6.35 (6.27–6.42)	0.77 (0.77–0.78)	88.38 (88.28–88.48)
Social studies	5.86 (5.78–5.94)	0.62 (0.61–0.63)	87.40 (87.29–87.51)
Historical & philosophical studies	5.85 (5.72–5.97)	0.24 (0.23–0.25)	85.46 (85.27–85.65)
Veterinary science	5.82 (5.25–6.39)	0.01 (0.01–0.01)	90.76 (90.05–91.47)
Mass communication & documentation	5.74 (5.58–5.90)	0.15 (0.14–0.15)	86.39 (86.15–86.62)
Physical sciences	5.71 (5.59–5.83)	0.25 (0.24–0.25)	87.72 (87.54–87.89)
Engineering & technology	5.53 (5.42–5.63)	0.33 (0.32–0.34)	90.60 (90.47–90.73)
Education	5.42 (5.31–5.52)	0.28 (0.27–0.29)	89.04 (88.89–89.19)
Biological sciences	5.08 (5.00–5.14)	0.62 (0.61–0.63)	87.52 (87.42–87.63)
Medicine and dentistry	5.03 (4.85–5.20)	0.09 (0.09–1.00)	91.36 (91.13–91.58)
Computer science	5.00 (4.89–5.10)	0.26 (0.25–0.26)	87.45 (87.29–87.61)
Business & administrative studies	4.40 (4.33–4.46)	0.55 (0.54–0.55)	91.67 (91.58–91.75)
Languages	3.25 (3.16–3.34)	0.16 (0.15–0.16)	88.11 (87.95–88.27)
Mathematical sciences	3.23 (3.09–3.37)	0.07 (0.06–0.07)	90.61 (90.39–90.84)
Law	3.14 (3.04–3.23)	0.13 (0.12–0.13)	90.34 (90.18–90.50)

a % of all students studying that subject area. ^b^% of all registered students across all subject areas.

*Note*. SpLD/ADHD = specific learning difficulties/attention-deficit/hyperactivity disorder; HESA = Higher Education Statistics Agency.

### HEI Type

We coded each institution according to whether it was: (a) a pre-92 university (all); (b) a pre-92 university excluding Russell Group universities; (c) a pre-92 Russell Group university; (d) a post-92 university; (e) an HEI which teaches only specialist subjects such as creative arts, performing arts, or agriculture. As Table 6 shows, a relatively higher proportion of students reported SpLD/ADHD at post-92 universities (5.41% of the total student body) than at pre-92 universities (4.37%); a higher proportion reported SpLD/ADHD at Russell Group universities (4.56%) than at non-Russell Group universities (4.21%). The highest relative proportion of students reporting SpLD/ADHD were at specialist institutions (12.22%).

**Table 6.** Institution Types Attended by Students With Reported SpLD/ADHD and Those With No Known/Reported Disability (Source: HESA).

	SpLD/ADHD		No known disability	
Institution type	Number	%^a^	Number	%^a^
Pre-92 university (all)	107,760	4.37	2,228,325	90.31
Pre-92 non-Russell Group university	57,890	4.21	1,234,055	89.84
Russell Group university	49,870	4.56	994,270	90.90
Post-92 university	160,090	5.41	2,648,825	89.51
Specialist institution	20,690	12.22	137,840	81.40

a Although percentages were calculated against total number of students within each group, only results for those with no reported disability or SpLD/ADHD are reported.

*Note*. SpLD/ADHD = specific learning difficulties/attention-deficit/hyperactivity disorder; HESA = Higher Education Statistics Agency.

Across all institutions, comparison was made between the top 10 (proportion of students with declared SpLD/ADHD) and bottom 10 (see Table 7). Amongst the top 10 institutions, 8 specialize in creative and performing arts, 2 in agriculture and veterinary science. Amongst the bottom 10 institutions, 6 offer a range of general HE courses, 1 specializes in pharmaceuticals, 2 in teacher training, 1 in science, technology, medicine, and business.

**Table 7.** Higher Education Institutions With the Most and Least Students Reporting an SpLD/ADHD (Source: HESA).

University	Type of courses provided	% of students ^a^
Royal Agricultural University	Agriculture & veterinary science	22
Conservatoire for Dance and Drama	Performing arts	18
Harper Adams University	Agriculture & veterinary science	16
Falmouth University	Creative arts	16
Royal Central School of Speech and Drama	Performing arts	16
Leeds Arts University	Creative arts	15
University of the Arts, London	Creative arts	14
Royal Conservatoire of Scotland	Performing arts	14
Rose Bruford College	Performing arts	14
Guildhall School of Music and Drama	Performing arts	13
Imperial College London	Science, technology, medicine, and business	3
University of Cambridge	General education	3
University of Sunderland	General education	3
Aston University	General education	3
University of Strathclyde	General education	3
Queen’s University Belfast	General education	2
The Open University	General education	2
St Mary’s University College, Belfast	Teacher training and liberal arts	2
Stranmillis University College	Teacher training	1
The School of Pharmacy	Pharmaceuticals	1

a Rounded to the nearest whole % in accordance with the HEIDI Plus Rounding Methodology.

*Note*. SpLD/ADHD = specific learning difficulties/attention-deficit/hyperactivity disorder; HESA = Higher Education Statistics Agency; HEIDI = Higher Education Information Database for Institutions.

## Discussion

This study sought to investigate the prevalence of SpLD/ADHD amongst first-year students in UK HEIs—the courses they selected and institutions they attended—in relation to their sex and ethnicity, to identify overarching patterns and trends. In the light of ongoing efforts to increase equality, diversity, and inclusion in HE, for example, through the work of Advance HE’s Equality Challenge Unit (https://www.advance-he.ac.uk/guidance/equality-diversity-and-inclusion/student-recruitment-retention-and-attainment/attracting-and-increasing-student-diversity), this study offers an important, systematic update on earlier UK figures. Unlike previous studies which have relied on HEIs providing data in response to researcher requests, the current study included data obtained directly and formally via HESA, providing data from almost 5.7 million students across 12 years.

### Prevalence of SpLD/ADHD in Relation to Sex and Ethnicity

Number of students reporting SpLD/ADHD increased from 17,510 in 2007/08 to 26,705 in 2018/9. Even within the context of increasing HE student recruitment, this represents a gradual increase in prevalence of reported SpLD/ADHD, from 4.08% (2007/08) to 5.57% (2018/19). This is higher than reported in earlier studies of UK students—0.46% in 1995 (Richardson & Wydell, 2003); 1.35% in 1999 (Singleton, 1999); 1.51% in 2001 (Richardson & Wydell, 2003, although these pre-2007/08 figures only included students with dyslexia, not other SpLD/ADHD); and 4.97% in 2012 (Richardson, 2015), but it is still lower than expected. The prevalence rate for dyslexia is around 10%, for dyspraxia it is around 5%, and for ADHD it is around 4% (British Dyslexia Association, 2018), but these difficulties frequently co-occur, such that approximately 60% of people with dyslexia are also dyspraxic, and approximately 30% also experience ADHD (John-Adubasim & Ugwu, 2019); up to 50% of people with dyspraxia also have ADHD (Goulardins et al., 2017). Figures from 2019/20 show that only 17.5% of UK secondary school pupils with special educational needs (SEN) requiring additional support entered HE compared to 47.5% of non-SEN pupils; those with social, emotional, and mental health needs (including ADHD) or specific learning difficulties (including dyslexia and dyspraxia) had a progression rate of 10.4% (GOV.UK, 2021). These figures, along with our own, indicate that, despite a move toward widening participation in UK HE via legislative changes (such as the Special Educational Needs and Disability Act [SENDA], His Majesty’s Stationery Office [HMSO], 2001), the offering of flexible entry requirements, greater learning support and accessibility, and reasonable adjustments (Pino & Mortari, 2014; Richardson & Wydell, 2003; Stampoltzis & Polychronopoulou, 2008), more needs to be done to understand why greater numbers of pupils with SpLD/ADHD do not progress to HE.

Within the population of first-year SpLD/ADHD students were approximately equal numbers of men and women. Historically, dyslexia, dyspraxia, and ADHD have been reported to be more prevalent in males than females (Gaub & Carlson, 1997; Quinn, 2018; Rutter et al., 2004) although ratios have varied depending on sample characteristics, extent of difficulties, and diagnostic criteria used. Some researchers have found that the male:female ratio increases with severity of learning difficulties (Arnett et al., 2017; Hawke et al., 2007; Quinn & Wagner, 2015), while a recent review identified proportional increases in females identified with learning difficulties over time (from 2015/16 to 2022/23), and as a function of phase of compulsory schooling (Daniel & Wang, 2023). These authors report an overall male:female ratio for learning difficulties in England of 1.27:1. These trends might explain the absence of observed sex difference in our students, who possibly have milder forms of learning difficulty having successfully accessed HE. Unfortunately, it is not possible to test for this, or to control for the fact that the data only include students whose difficulties had already been identified and reported at the time of university application.

While SpLD/ADHD occurs in all racial and ethnic groups, our data show greater relative prevalence among White and Mixed ethnicity students than amongst students who are Black, of Other ethnicity, or Asian. These findings are consistent with those reported elsewhere (UK adults: Richardson & Wydell, 2003; Strand & Lindorff, 2018; UK school children: Emerson, 2012; US children: Morgan et al., 2017; Odegard et al., 2020). Of particular note is the disproportional identification of SpLD/ADHD amongst Asian students, of whom only 2% self-identify as having SpLD/ADHD. Previous research has shown that Asian students are substantially less likely to be identified with SpLD/ADHD than are White students (Cooc, 2019; Dyson & Gallannaugh, 2008; Strand & Lindorff, 2018) even when age, sex, and socio-economic status are controlled for. There are several possible explanations for this: parents from Asian backgrounds may be less aware of SpLD/ADHD, and may perceive their children’s difficulties as stemming from insufficient effort or motivation. Where difficulties are acknowledged, parents may feel stigmatized and fear being judged; they may be unaware of available support, and where this is offered, parents may experience linguistic or cultural barriers to accessing it (Asghar et al., 2019; Mueller et al., 2012; Strand & Lindorff, 2018). Alternatively, it may be that Asian students with SpLD/ADHD are just less likely to apply to study at a higher level. Additional research is needed to differentiate between these possibilities.

### SpLD/ADHD and Subject Choice

Our results show that students with SpLD/ADHD are more likely to study creative arts and design (especially visual and performing arts), agriculture and related subjects, architecture, building and planning, subjects allied to medicine, social studies, historical and philosophical studies, physical sciences, and veterinary science than they are other disciplines. They are least likely to study medicine and dentistry, computer science, education, biological sciences, mathematical sciences, business and administrative studies, law, and languages. This is consistent with earlier findings (Longobardi et al., 2019; Richardson & Wydell, 2003; Shrewsbury, 2015; Stampoltzis & Polychronopoulou, 2008). The literature contains reports of high numbers of students with dyslexia and other SpLD in HE departments offering arts subjects, with some art and design schools reporting prevalence rates up to 30% amongst some cohorts (Holgate, 2015; Steffert, 1999). Other studies, including the current one, suggest that the figure is approximately 10–15% (see Wolff & Lundberg, 2002). Higher than expected numbers of individuals with SpLD are also reported in dance and drama (Oram, 2018; Royal Conservatoire of Scotland, 2018), design and the creative arts (Rankin et al., 2007; Wolff & Lundberg, 2002), architecture (Holgate, 2015; Royal Institute of British Architects [RIBA], 2023), nursing (Sanderson-Mann & McCandless, 2006; Wray et al., 2012), and agriculture (Smith et al., 2016).

Why are those specific courses more likely to be studied by SpLD/ADHD students? It may be that students with SpLD/ADHD preferentially apply to study subjects at the top of Table 5 because these reflect their strengths, interests, and career intentions. Many students with SpLD/ADHD study subjects characterized as practical, less linguistic, and multi-dimensional, mainly in the creative, visual, and performance arts, agriculture, and forestry. Kortering et al. (2010) reported that adolescents with SpLD were more likely to express a wish to enter the construction industry or law enforcement, while adolescents without SpLD expressed intentions to pursue careers in education, medical and health services, or business. Similarly, Gutman and Schoon (2018) found that compared to their non-SpLD peers, adolescents with SpLD were significantly less likely to aspire to have professional or managerial careers but more likely to aspire to have skilled or unskilled jobs. Alternatively, lower numbers of students with SpLD/ADHD studying law, mathematical sciences, medicine, and languages may reflect a lack of interest or ability in subjects that are heavily characterized by literacy and numeracy (Bacon & Bennett, 2013; Savolainen et al., 2008; Winner et al., 2001). Those who opt to pursue many of the more practical and creative subjects and occupations view them as involving less writing than other subjects and occupations (Holgate, 2015; Sanderson-Mann & McCandless, 2006; Smith et al., 2016). A third explanation is that students with SpLD/ADHD apply to the same courses as non-SpLD/ADHD students, but they disproportionately fail to secure places to study subjects listed toward the lower end of Table 5. Students with SpLD/ADHD are less likely than their non-SpLD/ADHD peers to achieve a “good” (first or upper second class) degree (Higher Education Funding Council for England, 2016; Richardson, 2015), so it might be expected that they are similarly less likely to achieve the “good” school exam grades they need to secure a university place to study law, medicine, mathematics, business, and computer science (see Chatzitheochari & Platt, 2019; Gutman & Schoon, 2018). Students with dyslexia and other SpLD have been found to demonstrate lower academic confidence and be less certain of achieving good grades relative to their non-SpLD peers (Brunswick & Bargary, 2022; Donato et al., 2022; Gutman & Schoon, 2018; Sumner et al., 2021) which is likely to influence their university applications.

### SpLD/ADHD and HEI Type

Proportionally more students with SpLD/ADHD attended post-92 than pre-92 universities. They were least likely to attend pre-92 non-Russell Group universities, and proportionally 2–3 times more students with SpLD/ADHD attended specialist institutions than other types of university. Richardson and Wydell (2003) reported a similar pattern across institution types although their percentages were slightly lower. This pattern may reflect the nature of the courses offered by the different institutions, with SpLD/ADHD students preferentially applying for the more vocational and creative subjects (potentially involving less reading, writing, and mathematics) offered by post-92 universities and institutions specializing in art, drama, music, and agriculture; in this way, these students’ strengths and interests may be driving both their choice of subject and HE provider. Alternatively, it may reflect a difference in accessibility of different HEIs: in general terms, pre-92 universities have tended to be more selective than post-92 universities, as indicated by the higher qualification levels of their applicants and entrants, their smaller proportion of entrants relative to applicants (i.e. their selectivity), and the smaller number of students they accept through clearing (Boliver, 2013; Raffe & Croxford, 2015). Therefore, they are less accessible to students with poorer school exam grades and lower academic confidence (Chatzitheochari & Platt, 2019; Donato et al., 2022; Richardson, 2015).

Post-92 universities have sought to widen participation in HE and attract under-represented groups, often from the local community, with nonstandard entry qualifications such as vocational qualifications (McCaig, 2015; Purcell et al., 2009). In contrast, pre-92 universities traditionally lean more toward “fair access” by offering places to disadvantaged but academically strong students (McCaig, 2015). Specialist HE providers also place greater emphasis on practical experience and demonstrable skills, via a portfolio of work, for example, as part of their entry requirements unlike traditional academic subjects at university; they also tend to offer foundation courses as a route into degree programs for those who lack standard qualifications (Higher Education Funding Council for England, 2015).

### Strengths, Limitations and Future Directions

This study sought to provide an analysis of data from almost 5.7 million UK-domiciled first-year foundation and first-degree students at UK HEIs over 12 years. It enabled us to produce a comprehensive account of students who reported SpLD/ADHD year on year, their sex and ethnicity, programs and subjects studied, and universities attended. While this method of data collection is comprehensive, it is not without challenges, and it presented our study with some issues and limitations. For example, in 2007, HESA started combining data on students with dyslexia, dyspraxia, and ADHD into a single category, so we are not able to provide a more detailed analysis of the specific type of learning need, and it is unknown the extent to which the academic decisions and difficulties of students with dyslexia and dyspraxia overlap with those of students with ADHD. The inclusion of a “two or more conditions” category in the HESA data means that some students with dyslexia, dyspraxia, and/or ADHD plus a co-occurring condition may have appeared here rather than in the specific learning difficulty category. We took the decision not to include students with “two or more conditions” in our analyses to avoid potentially counting students twice, but this may have resulted in some students not being counted at all and this is acknowledged. The data are reliant on self-report rather than formal assessment and, although it is common practice within educational research to identify students with learning difficulties in this way, it is a potential limitation because an objective demonstration of educational need is not available. Furthermore, students whose SpLD/ADHD are identified only after they have started at university would have been misclassified; it is not possible from the current data to gauge the extent or effect of this misclassification. In HESA’s data, students are classified as either having a difficulty/disability or not and there is no indication of severity. As SpLD/ADHD exist on a continuum, this large dataset is likely to include students with varying levels of dis/ability. It is not possible, therefore, to draw conclusions regarding whether the severity of SpLD/ADHD is associated with any of the variables studied here. Finally, in the HESA database, there are missing values—each variable includes an “unknown” category, and in some cases “information refused” (ethnicity). However, as a percentage of the total dataset, numbers of missing values are small.

One potential avenue for future research would be to conduct a more formal exploration of why students with SpLD/ADHD study these particular subjects, to tease apart the possibility that it is (a) out of interest, if students are actively pursuing more creative and practical courses that align with their strengths; (b) out of necessity, if students fail to gain a place on a preferred course; (c) out of fear of applying to a more competitive course that students perceive to be heavily language- or number-based. If the reason proves to be either from necessity or fear, then this might reasonably help to direct the focus of those employed to support and advise students as they progress through tertiary education, and those involved in making HE admissions decisions.

In summary, more students have declared SpLD/ADHD on their university applications year on year since 2007/08, both in actual terms and as a proportion of the student body. These students were most likely to be White or of Mixed ethnicity, and least likely to be Asian; they were most likely to attend a specialist institution or post-92 university, and least likely to attend a pre-92 university. Approximately equal numbers of men and women declared SpLD/ADHD, and these students were much more likely to be studying predominantly creative and practical subjects rather than more heavily language- and number-based ones.

## Notes

1. We suggest that this term used by HESA may not be the most accurate one. Dyslexia and dyspraxia are specific learning difficulties that directly affect an individual’s ability to receive and process information. Attention-deficit/hyperactivity disorder (ADHD) is not a specific learning difficulty although it makes it harder for individuals to concentrate, and thus to focus on learning. Therefore, for the remainder of this paper, we will use the term SpLD/ADHD when referring to students from the HESA dataset (post-2007/08) who report dyslexia, dyspraxia, or ADHD.

2. A period outside the normal application window when students, who may not have achieved the grades required to allow them to accept conditional offers from their preferred universities, have an additional opportunity to apply for university places which have yet to be filled.

3. These numbers broadly follow the ethnic breakdown of the United Kingdom based on the 2019 England and Wales Census data: 84.8% of people identified as White, 8% as Asian, 3.5% as Black, 1.9% as Other, 1.8% as Mixed (https://www.ons.gov.uk/peoplepopulationandcommunity/populationandmigration/populationestimates/articles/populationestimatesbyethnicgroupandreligionenglandandwales/2019)
